# Efforts to enhance the quality of life

**Published:** 2014-03-25

**Authors:** VL Purcarea

**Affiliations:** „Carol Davila” University of Medicine and Pharmacy Bucharest, Romania

 One of the affections which is characteristic to the modern world and which is constantly raising is diabetes. It represents a problem of public health, due to the high amount of the complications and the great costs associated to their treatment. This is why the efforts in this direction have become more and more sustained. 

 For 40 years, the Romanian Society for Diabetes, Nutrition and Metabolic Diseases (RSDNMD) has been keeping a long tradition as far as the attention given to diabetes in Romania is concerned. All the activities of the society have been concentrated on raising the quality of care and of life of the patients with diabetes. 

 The year 2013 has been a very productive one for the Romanian Society for Diabetes, Nutrition and Metabolic Diseases. At the national level, the society has kept on organizing the professional courses for the doctors involved in the diabetes field (professionals and resident doctors with the following specialties: diabetes, endocrinology, cardiology, nephrology, etc., but also family doctors, surgeons, neurologists, etc.), as well as the projects addressed to the public, such as the National Campaign “Control Your Diabetes”, which, this year, has taken place in Constanta and Cluj Napoca, an event which had as a major theme the prevention of diabetes and raising the awareness of precocious diagnosis and the prevention of diabetes complications. The 39th National Congress of the Romanian Society of Diabetes, which, during the 4 days of congress has reunited famous people in the field of diabetes from the whole world, among whom we should mention Andrew Bolton President of EASD, together with important personalities in the field of medicine in Romania. 

**Fig. 1:**
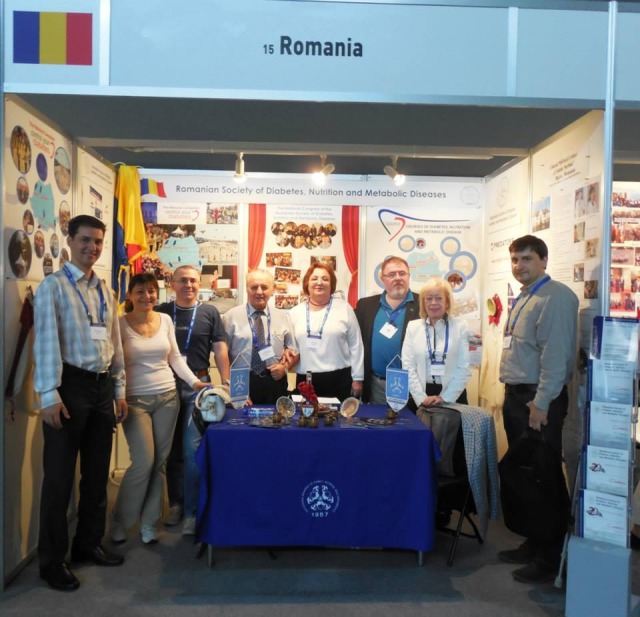
EASD 2013 International Congress in Barcelona

**Fig. 2:**
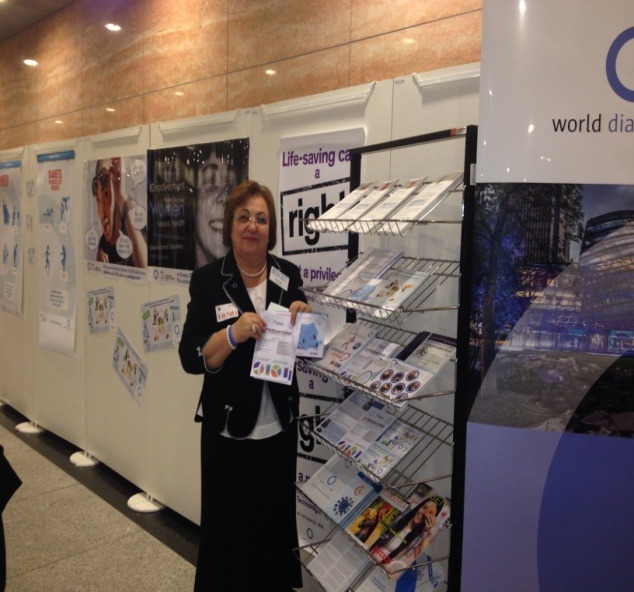
The Romanian Stand in the building of the European Parliament

Just like every year, the Romanian Society for Diabetes, Nutrition and Metabolic Diseases (RSDNMD) has been present to some important International Congresses such as the one of the European Society of Diabetes (EASD) in Barcelona, the one of the American Association of Diabetes (AAD) in Chicago, the one of the International Federation of Diabetes (IFD) which took place in Melbourne, events in which the Society has distinguished itself through scientific papers and stands. 

 On the World Day of Diabetes, RSDNMD has been present at a “breakfast meeting” organized at the European Parliament in Brussels with the help of the president – Professor Maria Mota. The discussions have been moderated by Baroness Sarah Ludford, in the presence of Michael Hirst, the IFD president and of Joao Manuel Valente Nabais, the IFD president in Europe. The speakers in this event were Elodie Besnier, IFD officer and Maria Mota. Elodie Besnier, who is currently involved in studying the Access to Quality Medicines and Medical Devices for Diabetes Care in Europe, has presented the results of the European study. 

**Fig. 3:**
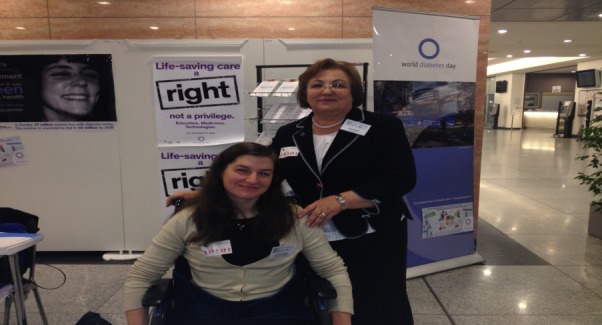
Elodie Besnier together with Maria Mota

**Fig. 4:**
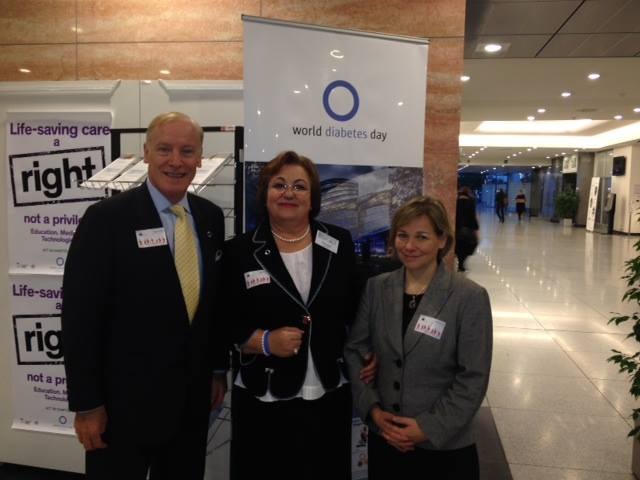
Maria Mota together with the IFD president

The presentation of Professor Maria Mota has been centered on the 3 aspects studied by IFD Europe: the accessibility to medication of the patient with diabetes (accessibility), the availability of the medication (availability), and the affordability of the costs (affordability) in our country. The efforts of RSDNMD to improve the diabetes network in Romania have been very much appreciated by the participants. 

Both children and adults with diabetes from European countries participated in the discussions. They have presented their situation as patients. Moreover, professor Jean Philippe Assal, the parent of therapeutic education of patients in Europe was also present at the discussions. 

 Parallel with this meeting, IFD Europe in collaboration with the European Parliament has organized an exhibition in the building of the Parliament, where diverse educational materials regarding diabetes were exposed. 

 One of the most important world events regarding diabetes was the International Congress of the International Federation of Diabetes (IFD), which took place this year in Melbourne at the beginning of December (2-6 December 2013). Just like every year, Romania had representatives at the highest level, our delegates participating with papers at the Congress, as well as in the international programs which IFD has gotten us used to already (“Young Leaders in Diabetes” – YLD), as well as in a new project which IFD has launched in 2013, Parliamentary Champions for Diabetes forum. 

 Initiated in 2011, the YLD program, in which youth with diabetes all over the world take part, is a key program which proposes to raise the level of information regarding diabetes especially for the youth, the implementation of prevention and education programs and last but not least, the elimination of discrimination at world level. Among the 130 youngsters from 73 countries, who have reunited this year in Melbourne there were also two youngsters from Romania, Matyas Daboczi and Cristina-Maria Petrut. The two of them took part in the training organized by important speakers in the world of diabetes, among whom the IFD president, Michael Hirst. The issues debated were the problems each community dealt with concerning diabetes and the bases of new future projects for the improvement of the diabetes network at a world level have been established. 

**Fig. 5:**
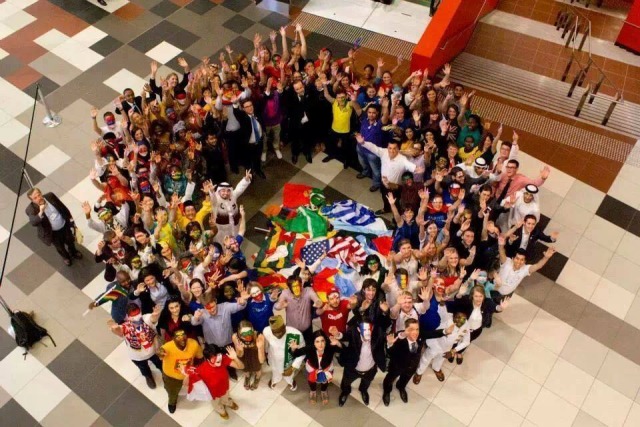
Young leaders in diabetes

“Parliamentary Champions for Diabetes forum” represented the novelty this year. Parliamentarians from 50 countries have taken part in this event; they had a sustained activity in the field of diabetes. Moreover, the IFD president, the European Commissioner for Health, Tonio Borg, the First Lady of South Africa, almost all the members of the Government of Victoria – Australia and representatives of the Federal Government of Australia have been present in the event. On this occasion, Melbourne Declaration has been adopted and signed by all the members present.

**Fig. 6:**
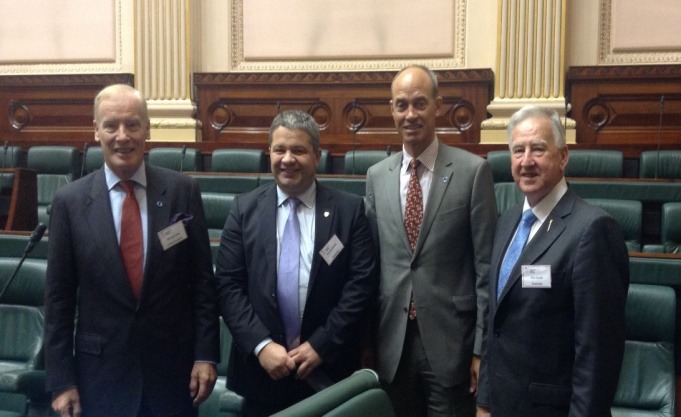
Florian Bodog together with Sir Michael Hirst,Guy Barnett, Ambassador of Australia for Diabetes and Ken Smith, member of the Parliament of Victoria

**Fig. 7:**
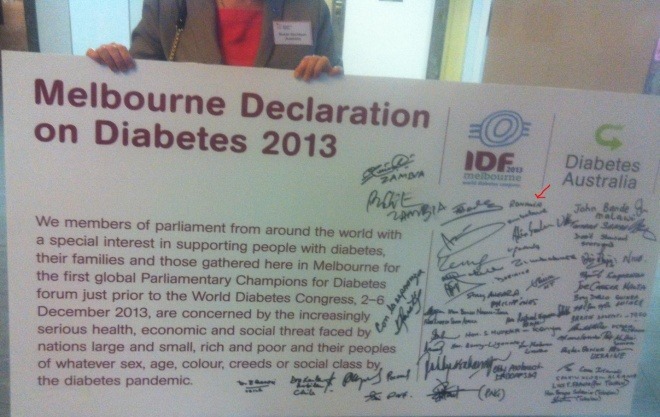
7 Melbourne Declaration

Through this Declaration, the social, economical and medical threats due to the pandemic raise of diabetes are recognized. The members who signed the Declaration have engaged to bring diabetes on the political agenda of each country, so that more prevention and precocious diagnosis activities will take place.

 We are proud that the first member of the Parliament who has signed the Declaration is a Romanian Senator, Assoc. Prof. Florian Bodog, MD, Secretary of the Health Commission of the Senate of Romania. The IFD Congress in 2013 had an enormous success, the 10.000 participants discussing about real interest problems not only for the diabetes field, but also with great impact at the level of the entire population, taking into account the epidemic raise which diabetes has had in the last couple of years. Among the scientific papers presented this year in the Among the scientific papers presented this year in the Congress, there were also papers from our country, the Romanian Society for Diabetes, Nutrition and Metabolic Diseases officially presenting data from the broad epidemiologic study, PREDATORR, which took part in Romania this year. 

**Purcarea VL, MD, PhD, Eng**

**Executive Editor**

